# Department Hierarchy and Letters by the Younger Generation

**DOI:** 10.31662/jmaj.2025-0461

**Published:** 2025-11-21

**Authors:** Shigeki Matsubara

**Affiliations:** 1 Department of Obstetrics and Gynecology, Jichi Medical University, Tochigi, Japan; 2 Department of Obstetrics and Gynecology, Koga Red Cross Hospital, Koga, Japan; 3 Medical Examination Center, Ibaraki Western Medical Center, Chikusei, Japan

**Keywords:** departmental hierarchy, letter, paper, professor, resident

## To the Editors,

Koga’s Letter ^(1)^, written by a Japanese resident physician in the United States, is significant. While complementing Saeki’s article ^(2)^, it emphasizes that Japan’s academic hierarchy may discourage younger physicians―particularly residents―from independently authoring Letters.

As Koga notes, “resident-only” Letters appear rare. I therefore examined how many were written by younger physicians at a Japanese medical university. As a retired obstetrics and gynecology (OBGYN) professor familiar with departmental hierarchies, I focused on OBGYN. On September 17, 2025, a PubMed search of the Japan Society of Obstetrics and Gynecology journal (“J Obstet Gynaecol Res” and letter) yielded 252 Letters (1999-2025). Excluding my own, 22 Letters originated from the same Japanese university department; professors’ names were absent in only two―both single-authored papers by writers in their forties and fifties. Notably, no Letters were authored by residents without a professor’s name.

Letters from abroad were not examined due to technical limitations, and the small sample cautions against firm conclusions. I do not claim that a professor coauthoring a resident’s Letter is inappropriate; a resident may write their own thoughts while a professor polishes them, which can be welcome. Nevertheless, the absence of independent “residents’ Letters” struck me.

Indeed, during my 18-year professorship, no residents in my department wrote Letters (opinion pieces). I assumed many residents focused on case reports or original articles, or were not motivated to express themselves independently. Although I dislike academic hierarchy and made this clear to my staff, I may have created an atmosphere where “the professor’s name should appear.”

I have suggested readers wish to hear residents’ perspectives in Letters―voices reflecting a uniquely younger sensitivity ^(3)^. Koga captured this perfectly: his keen, youthful perspective clearly identified the barriers preventing such contributions.

I have two humble requests. First, journals could consider a “Younger Voice” section, accepting only residents’ opinions, similar to “Patient Voice” or “Student Corner” in some journals. Second, professors should read Koga’s Letter: it may spark broader discussion among senior faculty―key figures in this issue.

Still, I respectfully disagree with Koga. Letter writing is not so easy ^(3), (4)^. While it may take less time than original research, composing a Letter based solely on one’s thoughts or convictions demands deep insight. A Letter’s quality is independent of the author’s age. Undoubtedly, Koga produced an excellent Letter. Seasoned physicians should encourage ambitious younger colleagues to write such Letters independently.

## Article Information

### Author Contributions

Study design, manuscript writing, editing, approval, and ICMJE authorship criteria met: Shigeki Matsubara.

### Conflicts of Interest

None

### Ethics Approval

Jichi Medical University does not require Institutional Review Board approval for this type of study. Preservation of patient anonymity and informed consent for reporting are not applicable.

### Patient Anonymity

Not applicable.

### Informed Consent

Not applicable.

### Data Availability Statement

Data sharing is not applicable to this article, as no new data were created or analyzed in this study.
